# The importance of school in the management of Myalgic Encephalomyelitis/Chronic Fatigue Syndrome (ME/CFS): issues identified by adolescents and their families

**DOI:** 10.1111/hsc.13942

**Published:** 2022-08-22

**Authors:** Philippa Clery, Catherine Linney, Roxanne Parslow, Jennifer Starbuck, Amanda Laffan, Jamie Leveret, Esther Crawley

**Affiliations:** ^1^ Centre for Academic Child Health University of Bristol Bristol UK; ^2^ Royal United Hospitals Bath NHS Foundation Trust Bath UK

**Keywords:** adolescent health, child & adolescent health, fatigue syndrome chronic, paediatrics, parents vs schools

## Abstract

Paediatric Myalgic Encephalomyelitis (ME)/Chronic Fatigue Syndrome (CFS) is a disabling condition. Schools play a key role in adolescents' experiences with managing ME/CFS. However, little is known about the experiences of adolescents with ME/CFS (and their families) in schools. This paper is an incidental qualitative study, which combines data from two independent ME/CFS studies: study 1 researched ethnic minority adolescents with ME/CFS; study 2 explored Acceptance and Commitment Therapy for adolescents with ME/CFS who had not recovered after one year. Participants included: adolescents with ME/CFS; their families; and medical professionals (ME/CFS specialists and non‐specialists). Adolescents, their families, and ME/CFS medical professionals were recruited from a UK specialist paediatric ME/CFS service. Non‐ME/CFS medical professionals were recruited from the same region. Semi‐structured qualitative interviews and focus groups were undertaken. Participants' views on schools from each study were combined and thematic analysis was used to identify themes. Fifteen adolescents with ME/CFS (11–17 years old), sixteen family members, and ten medical professionals (GPs, school nurses and ME/CFS specialists) were interviewed. Four key themes were found: (1) adolescents identified school was important for aiding ME/CFS recovery, especially educationally and socially; (2) families described varying levels of support from schools and local authorities with help managing ME/CFS – some described significant practical and emotional difficulties to accessing education, whereas others recounted examples of positive supportive strategies, particularly when teachers had previous experience or knowledge of ME/CFS; (3) parents thought three‐way communication between schools, healthcare and families could improve support; (4) participants felt schools were an appropriate place for knowledge building and raising awareness of ME/CFS amongst teachers and pupils, to aid improved supportive measures. In conclusion, this paper provides rich data that highlights the importance of education and the realistic fears and hurdles for adolescents with ME/CFS remaining engaged in education and the impact on their future. Some families described positive strategies in school, which were viewed as helpful to manage ME/CFS in the classroom. These strategies could be implemented alongside knowledge building initiatives and improved communication between healthcare and education. There is a need to further investigate useful strategies and determine how teachers can be best supported in implementing them.

**What is known about the topic**

- Paediatric Myalgic Encephalomyelitis (ME)/Chronic Fatigue Syndrome (CFS) can negatively impact on school attendance and educational achievement.
- Liaison between healthcare and schools is important for recovery from ME/CFS.
- There is little qualitative research on the experience of adolescents with ME/CFS in schools.

**What this paper adds**

- This paper highlights the importance of education and the realistic fears and hurdles for young people with ME/CFS remaining engaged in education and the concerns about the impact on their future.
- Some adolescents with ME/CFS experienced difficulties with school, which they felt negatively impacted on their future potential whereas others described supportive experiences with schools recognising their individual needs and built their confidence inattending school.
- Adolescents and their families felt school support was particularly good when teachers had knowledge of ME/CFS, therefore there is a need to further investigate useful strategies and determine how teachers can be best supported by healthcare professionals in implementing them.

## 1 INTRODUCTION

Paediatric Myalgic Encephalitis (ME)/Chronic Fatigue Syndrome (CFS) is a disabling and common condition (prevalence estimates 0.55–0.98% [Lim et al., 2020]) with symptoms including pain, cognitive dysfunction and post‐exertional malaise (NICE, 2007). Young people with ME/CFS in specialist ME/CFS services attend a median of two days of school per week (Crawley & Sterne, 2009; Davies & Crawley, 2008; Knight et al., 2018) and mean time absent from school is one year (Rangel et al., 2000).

Attending school is important for educational and psychosocial development in childhood and adolescence (Eccles, 1999), and childhood chronic illness can increase the risk of poorer educational and employment outcomes later in life (Maslow et al., 2011). The World Health Organisation recognises the importance of secondary education in their global strategy for adolescent health (World Health Organisation, 2016) and under UK law, suitable statutory education should be available to children with chronic health conditions (Department for Education, 2013, 2015).

Adolescents with ME/CFS place school at the centre of their recovery (Parslow et al., 2015), as part of a broader definition of “rebuilding life” in terms of educational and social reintegration (Harland et al., 2019; Jelbert et al., 2010; Rowe, 2019). They consider the relationship between healthcare and school to be pivotal in achieving this (Parslow et al., 2015). Most research to date focuses on quantitative outcomes of school absenteeism and academic achievement (Sankey et al., 2006; Tollit et al., 2018), failing to detail the wider experiences of adolescents with ME/CFS when engaging with schools and the education system. One study investigated the role of primary schools in the care of young children with ME/CFS (Brigden et al., 2020), but is limited in its application to adolescents. There is little qualitative research about adolescents' views on managing ME/CFS in secondary education.

This paper provides a descriptive account from adolescents with ME/CFS, their parents/carers, and medical professionals, of their experiences managing ME/CFS within the secondary education system. This multi‐perspective view was taken to understand how schooling affects those with ME/CFS. This paper aimed to demonstrate the importance of education and explore how adolescents with ME/CFS can be better supported in school.

## 2 METHODS

### 2.1 Recruitment

This paper is an incidental qualitative study, which combines data about views on schools from two independent qualitative studies. Study 1 interviewed community ‘influencers’, non‐ME/CFS medical professionals, and adolescents with ME/CFS and their parents who identified as from an ethnic minority background, with the aim of improving access to treatment. Study 2 interviewed adolescents with ME/CFS, their parents, and ME/CFS medical professionals, to explore Acceptance and Commitment Therapy for adolescents with ME/CFS with persistent symptoms after 12 months of treatment.

All adolescents, parents and ME/CFS medical professionals were recruited from one UK specialist paediatric ME/CFS service. Other medical professionals were recruited from the same region. Convenience sampling was used. Table 1 shows eligibility criteria. Eligible participants were given information leaflets (face‐to‐face or emailed) and provided consent to be contacted, or were directly consented into the study, by each study's lead (CL, PC).

**TABLE 1:** Eligibility criteria for study 1 and study 2

Study 1	Study 2
Inclusion criteria	
Adolescents diagnosed with ME/CFS, aged 11–17 years old, and who identify as ethnic minority	Adolescents diagnosed with ME/CFS, aged 11–17 years old, and not recovered after 12 months of treatment
Parents were eligible if their child was eligible and consented to participate	Parents were eligible if their child was eligible and consented to participate
Non‐ME/CFS medical professionals from GP practices and school nurses	Medical professionals were eligible if they worked in the ME/CFS service
Exclusion criteria	
Participants with severe ME/CFS	Participants with severe ME/CFS

### 2.2 Data collection

CL and PC (both female) conducted semi‐structured interviews and focus groups for study 1 (March 2019–January 2021) and study 2 (February–September 2020), respectively. CL and PC independently produced topic‐guides for each participant group (Appendix S1) for their respective studies, with input from an experienced qualitative researcher (RP) and clinician (EC). Participants were given interview location options: the university, clinic, their home, or over Skype. Due to the COVID‐19 pandemic, all interviews from March 2020 were over Skype. Adolescents and parents were asked to be interviewed separately but given the option of being together. No interviews were repeated.

### 2.3 Data analysis

Interviews were recorded on an encrypted voice recorder and transcribed verbatim. Identifiable information was removed.

Both studies used thematic analysis (Braun & Clarke, 2006) to inductively code data. CL and PC analysed their own studies' transcripts independently by iteratively and repeatedly reading transcripts, annotating with draft ‘codes’, which were then grouped into broader ‘themes’ using Nvivo (QSR International Pty Ltd, 2015, 2018). CL and PC (and RP, JL, AL and JS) double‐coded transcripts for the independent studies and it became apparent there were overlapping themes about schools. Data from each study pertaining to schools were therefore combined to create a new dataset. Step three of Braun and Clarke (2006) was revisited by CL and PC to collate codes into potential new themes and sub‐themes. These were then reviewed and defined to produce the thematic summary. Any divergent views or contradictions between studies or between participants were highlighted and discussed with RP and EC. The descriptive account with illustrative quotes was written jointly by CL and PC and is presented in the results section of this paper.

### 2.4 Ethics

Ethical approval was granted by the NHS Research Ethics Committee (Study 1: 18/SW/0120; Study 2: 19/L0/1979).

## 3 FINDINGS

### 3.1 Participants

Forty‐one participants were interviewed.

Eleven participants were from study 1: three adolescents (aged 11–16 years, median 13.5; all female), five parents/carers (all female), three non‐ME/CFS medical professionals (GPs and schools nurses). Five families declined to take part; one did not meet eligibility criteria, two were uncontactable, and two declined due to COVID‐19 stresses. All adolescents chose to be interviewed with their parent/family member (one face‐to‐face (parent and child), three over Skype (one parent only; one parent and child; and one two family members and child)). Two medical professionals were interviewed face‐to‐face, one over Skype. Interviews lasted 20–50 min. Community ‘influencers’ interviewed did not talk about schools.

Thirty participants were from study 2: twelve adolescents (aged 12–17 years, median 15.5, ten female), eleven parents (ten female), and seven ME/CFS medical professionals (clinicians, occupational therapists, physiotherapists, and clinical psychologists; six female). One adolescent and their parent declined to take part due to life stressors. Two parent–child dyads chose to be interviewed together, the rest individually (two face‐to‐face and ten over Skype). Five ME/CFS medical professionals took part in a face‐to‐face focus group; two were interviewed individually. Interviews lasted 30–110 min.

### 3.2 Themes

Four key themes were identified (Table 2). Findings are presented with illustrative quotes from participants to ensure their voices are represented. Quotes begin with “S1” or “S2”, indicating which study (1 or 2) the participant is from, followed by “A”, “P” or “MP”, indicating adolescent, parent or medical professional, respectively.

**TABLE 2:** Key themes of the experiences of adolescents with ME/CFS of the UK schooling system, grounded in responses from all participants

Theme	Sub theme/s
1. Importance of School	*1.1. Missing school results in stress and anxiety around lack of education and lost social connections* *1.2. School: symptom or cause?* *1.3. Managed return to school aids recovery and avoids relapse*
2. Levels of Support	*2.1 Experiences with schools and local authorities* *2.2 The role of diagnosis and understanding in accessing support* *2.3 Supportive school strategies recognise individual needs and build confidence*
3. Three‐way communication between families, schools and health services is important	
4. Building awareness and ‘re‐educating’ in schools	

#### 3.2.1 Importance of school

All adolescents, parents and medical professionals discussed the importance of school in helping adolescents with ME/CFS. This first theme explores: missing school results in stress and anxiety around lack of education and lost social connections; is school a symptom or cause of ME/CFS?; managed return to school aids recovery and avoids relapse.

##### Missing school results in stress and anxiety around lack of education and lost social connections

Attending school was important to all adolescents. Many adolescents found missing school stressful, particularly “when things [got] really important” (S2‐A15) as they worried about catching up on work, which created additional stress. Parents said adolescents with ME/CFS tended to be “very bright” (S1‐P02) so being unable to attend school was particularly “devastating” (S2‐P06) and a “very dark time” (S2‐P13). Adolescents described a sense of loss and parents reflected how “going to school is such a big thing” (S2‐P06) for their children: “she thrives on her academic achievements, being with her friends…that's what she really was about” (S2‐P15).

Parents and adolescents were concerned that missing school would result in insufficient education. They worried about the long‐term impacts this could have on future career opportunities. Adolescents hoped they would “recover enough to live the life [they] want and go to university and have a job” (S2‐A13) but some were less hopeful: “I doubt I'll get a job, I'll probably end up, you know, just a nothing” (S2‐A18).

“I don't think [child] will get to university now[…]their life choices are taken away from them because they're not being educated and really every child should be educated.” (S2‐P16)

Adolescents in both studies also discussed losing social connections at schools. They found “it really hard to make friends” (S2‐A12) or “lost a lot of friends” (S2‐A11) because of school absence. “Need[ing] days off” or “go[ing] early” (S1‐A03) meant fewer opportunities to maintain old friendships or make new ones. ME/CFS professionals commented how adolescents worried “everyone's going to hate me because I've been out for six months, no one's going to be my friend” (S2‐MP1). This was compounded by having to explain or justify to peers why they were not in school. Most felt their friends did not understand ME/CFS and perceived them as “lazy” (S2‐A11).

“My friends just don't understand what [ME/CFS] is and they're always constantly asking me ‘why are you never in?’ or like ‘what's going on?’” (S1‐A03)

Some adolescents attributed their low mood and anxiety to missing school and losing friends. Adolescents valued seeing their friends, so being “taken away from peers … made life even worse” (S2‐P12) and caused them to be “quite down” (S2‐A16.1). One participant described having a panic attack upon returning to school because of prolonged absence.

##### School: symptom or cause?

Families had divergent views on whether the first symptoms of ME/CFS manifested as missing school, or whether stresses around school contributed to developing ME/CFS. One parent said they first noticed something was wrong when their child was not able to attend school:

“I noticed that she was missing school because I just couldn't wake her up in the morning.” (S1‐P03)

Other families felt that school, particularly exams, contributed to developing ME/CFS. One adolescent felt that the pressure of SATs (Standard Attainment Tests) caused her overwhelming stress and anxiety that contributed to her developing ME/CFS. One parent felt exams should be delayed, allowing “a child to be a child at primary school” (S2‐P06). ME/CFS specialist medical professionals also recognised that ME/CFS symptoms tended to improve when there was no exam pressure:

“It's often school that's the issue because I see so many [children] not recovering within a year, and as soon as they've done their GCSEs, there's a sudden, they start getting better.” (S2‐MP14)

##### Managed return to school aids recovery and avoids relapse

All participants felt returning to school was important for improved mood, educational opportunities, and social connectedness, which in turn aided recovery:

“Be[ing] able to go back to school properly has helped [recover]. And I've got some good, good friends[…]having the social as well as just the school bit has really helped.” (S2‐A12)

However, there were divergent views from adolescents and parents about managing the return to school. A few adolescents were not anxious about returning to school, had “always wanted to go back to school” (S2‐P04) and were adamant to return full‐time because they did not want to even “miss one day at school” (S2‐A02). However, the majority of adolescents were anxious about returning to school and described feeling “[they'd] never be able to go back to school” (S2‐A04), so valued a part‐time graded return: “I was thinking oh gosh I don't know if I'm gonna go back [to school]…I didn't go back full time…I feel a bit more positive just taking it day by day” (S2‐A11). Medical professionals commented that the “unpredictability [of the school day] draws on so much energy” (S2‐MP14), which could be why most adolescents found it helpful to establish a routine with the same teachers and friends every day to support building relationships and appreciated help from the ME/CFS service “managing hours at school” (S2‐A13) to achieve this.

“I go to college on [days and times] and I have a day off on [day]. I've been so much better since and I think being put on much more of a routine has helped.” (S2‐A05)

Parents also valued the ME/CFS service for “input in manag[ing] daily activities and school holidays” (S2‐P15). Most parents thought a phased return to school was necessary and cautioned that “pushing and pushing” (S2‐P02) to attend school was unsustainable. One parent believed it contributed to an exacerbation of their child's ME/CFS: “she couldn't do it…she was [back] at home because she couldn't cope” (S2‐P02). Most adolescents and parents thought it was better to attend school at a significantly reduced level than push to the point of being unable to attend altogether. Medical professionals agreed school should not be “pushy” (S2‐MP14) or “go at a speed that the young person is not able to go at” (S2‐MP14).

#### 3.2.2 Levels of support

Families and medical professionals talked about varying levels of support from school. Some felt there was a lack of support or described difficulties accessing education services, whereas others described supportive experiences.

##### Experiences with schools and local authorities

Many families described perceived difficulties around accessing education in the context of ME/CFS. Some parents had difficulties engaging with their child's school or local authority and “were really disappointed with [their] school” (S2‐P11) and their handling of the situation. Some described it as a “battle” (S2‐P12) or a “nightmare” (S2‐P13) to get their child “some sort of [legal] right to access education” (S2‐P13) and one parent described frequently “burst[ing] into tears” (S2‐P13) with frustration. Some adolescents felt their schools “didn't even try to understand” (S2‐A11), which parents thought “add[ed] a massive amount of stress to the child” (S2‐P16).

Parents described their difficulties accessing educational support from school and local authorities. For example, some had “trouble…with school because they kept losing the paperwork” (S1‐P05) for a “My Care Plan” (S2‐A13) or others said “school hadn't applied” (S2‐P02) for educational needs testing. Adolescents described being “promised a tutor[…]which never materialised” (S2‐P18) and that “[schools] would say ‘oh we'll get teachers to send you some work’, which they never did” (S2‐A13). Parents felt their children “slip[ped] through the system” and were “passed backwards and forwards” (S2‐P13) between responsible authorities. Some parents felt they spent a lot of time “firefighting admin” (S1‐P01) and chasing people with a “hideous amount of phone calls and emails” (S2‐P13).

A common perception amongst parents and adolescents was that “schools [are] so focused on attendance” (S1‐P01), which was incongruous with the parents' focus on “dealing with a child who was poorly” (S1‐P02) and trying to “get on with the diagnosis” (S1‐P01). A few adolescents described that school could be “pushy” (S2‐A06) with attendance and “made it really difficult for [the child] to comply” (S2‐P12) with the reduced timetables recommended by the ME/CFS service. Two families discussed how schools “complained that [their child's] attendance wasn't high enough” (S2‐P12) and stated that this led to the child not getting “rewards trips, or the cakes or the ice lollies” (S2‐P12), leaving the child feeling “punished” (S2‐P12) as a result.

“The teachers are very much attendance oriented so if she has time off they're very much ‘right well you don't get to take part in raffles at school, you don't get to go on trips because of your attendance is bad’.” (S2‐P02)

One parent described being “accused of not sending [their] child to school” (S2‐P16). Three other parents discussed experiences of “fines [they've] had to pay” (S1‐A01) or being “threatened by the local authorities” (S1‐P02), which they attributed directly to their child's poor school attendance due to ME/CFS:

“They would complain that her attendance wasn't high enough so they took us to the local authority and tried to have us prosecuted.” (S2‐P12)

However, families recognised that schools “have got their own pressures” (S2‐P15) to prioritise attendance and exam results “so they can sell themselves” (S2‐P17) and “meet strict targets for funding” (S2‐P06). These competing priorities between attendance versus wellbeing left parents feeling their child was “just a place in the school [for] funding” (S2‐P06).

##### The role of diagnosis and understanding in accessing support

Both studies found divergent views about whether increased support was provided after a ME/CFS diagnosis was received, or whether support remained lacking despite providing medical evidence.

One family felt school were more supportive once their child received a diagnosis and had appropriate medical evidence: “The minute [school] got the letter you know they were, ‘we're aware of this and will do whatever we can to help’” (S1‐P03). For many families, support from the ME/CFS service was integral to feeling validated, and facilitated the school engaging with ME/CFS treatment plans. Parents felt they did not have the authority, without help from the ME/CFS service, to enforce rest recommendations: “you need other people to tell the school what you're doing and the reasons behind it” (S2‐P04). For one family, recommendations from the service led to the implementation of rest rooms and frequent breaks, and another family described not receiving help until the service intervened:

“*without seeing somebody like [psychologist] the school wouldn't listen to me at all. [They] wrote letters to school and even has come all the way out to visit her school um to talk to the teachers about her and things to help*.” (S2‐P02)

In contrast, many families described no increased educational support following diagnosis “no matter how much medical evidence” (S2‐P12) they provided. These participants thought schools “didn't really believe [the adolescent was unwell]” (S2‐A11) or that “there [was anything] physically wrong with them” (S1‐P02) because medical tests were normal.

“I would go to school and all the teachers and all the students would be so confused as to what was wrong with me.” (S2‐A11)

Some parents theorised this might be because teachers had not come across ME/CFS before, and even if they had, it could be difficult to understand how to manage it because individuals' needs differed. Others attributed it to “higher ups” (S2‐P06) in local authorities who lacked knowledge of ME/CFS needs.

“*I think there's always a lack of understanding and I think that partly is because it affects each child differently so one recommendation isn't suitable for another and that's quite hard for people who aren't health professionals to understand.”* (S2‐P15)

##### Supportive school strategies recognise individual needs and build confidence

Of the 14 families interviewed, five described that their school had been helpful in supporting their ME/CFS in the classroom setting. Some families described specific strategies they found useful and an additional two families offered suggestions for strategies that could be helpful.

Four families who described their school had put some useful strategies in place, noticed that where teachers “ha [d] worked with other children with chronic fatigue” (S2‐P18) or were “educated with the condition” (S2‐P05), their children felt more supported. They said teachers with experience of ME/CFS recognised when adolescents needed a break and were able to explain ME/CFS to other students: “the teachers got [the class] aside and just explain [ed] it to [the students], it was really good” (S1‐P04). Adolescents described feeling “fortunate” (S1‐A03) and “lucky” (S2‐P05) to have teachers like this. One parent suggested there should be a ME/CFS lead “responsible for the welfare of the children with CFS” (S2‐P15).

“It's been really good and her tutor, he knows all about ME because he had a child with it so that's been really helpful, so he knows the signs and has told her to go home when she's drained and things.” (S2‐P05)

Examples of useful strategies that familes spoke about included: opportunities for rest (such as “nurture rooms where you can just go if you feel too tired” (S1‐A03)), being allowed to leave class “us [ing] exit passes” (S2‐P02); a slower pace in lessons; “part time timetables” (S2‐A02) to reduce overall time at school or not do physical education (P.E.), which helped prevent ‘boom and bust’ cycles of fatigue (a key goal of ME/CFS treatment).

“School has been really good and she's got various little cards that she can use if she needs to exit a lesson or, you know, ‘take five’, that sort of thing[…]the um timetable that she's following, where um she can go to a particular quiet area that's designated um and um either work quietly, or rest.” (S2‐P15)

Two adolescents mentioned that college was “so much better” (S2‐A05) in comparison to secondary school because it provided this autonomy and flexibility with reduced timetables or online courses being “way less intense, which [they] needed” (S2‐A13). One family “decided [they] wouldn't send [their child] to school” (S2‐P11) due to more flexibility home‐schooling with online resources and working at their own pace “as and when, when [their child] felt well enough” (S2‐P11).

Participants particularly valued when their individual needs were not just permitted, but actively supported and encouraged. They described how this built their confidence to manage ME/CFS symptoms at school. Some parents noted that if adolescents knew school was supportive of them to take breaks, they were more willing to return to school. If support was not provided, “it will only end up that [the young person] won't go [to school]” (S2‐P02) at all, for fear of not being allowed to rest when needed.

One parent suggested that to tackle the issue that their child was “not being educated” (S2‐P16) when physically unable to attend school when unwell, that there could be increased access to learning through private tutors, or “schools record [ing] the lesson [so pupils] could access the lesson from home” (S2‐P16). This parent commented that the distance learning set up by schools during the COVID‐19 pandemic could make schools better equipped to support those with chronic health conditions to work from home in the future:

“You know, how quickly did they set [COVID‐19 distance learning] all up?[…]There should be a very quick system in place where the school, either send out tutors, or they put something out or record the lessons in some way that the children can access their lessons later in the day. I feel really strongly about that.” (S2‐P16)

#### 3.2.3 Three‐way communication between families, schools and health services is important

**FIGURE 1:**
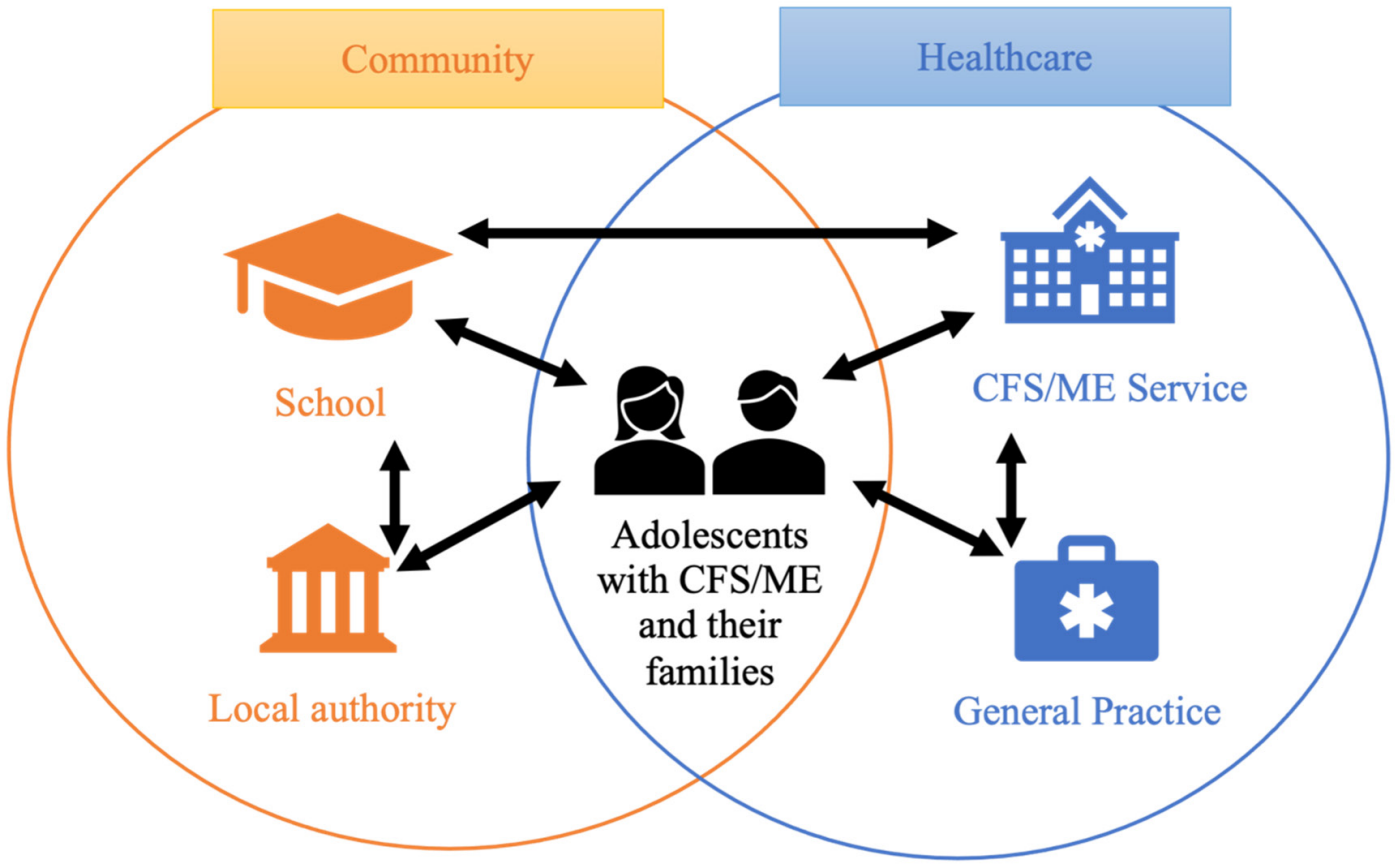
A model for integrated care between health and education systems for adolescents with ME/CFS and their families.

All participants identified problems with communication between the key professionals involved in the care of adolescents with ME/CFS. Three‐way communication between parents, schools, and healthcare services was thought to be integral (Figure 1).

Non‐ME/CFS medical professionals felt there was “pretty disparate” (S1‐MP02) communication between schools and primary healthcare (GPs). They commented there was a lack of “screening of general health problems” (S1‐MP02) in school and that schools were referring to GPs “just because of poor attendance” (S1‐MP02). They suggested school nurses would be well‐placed to aid diagnosis by making direct referrals to GPs or even ME/CFS services “rather than putting the [extra] barrier of saying you have to go to the GP” (S1‐MP03).

Communication from schools to the service was also important *after* diagnosis, because “fatigue will be affecting school performance” (S1‐MP03) so medical professionals thought it would be “one of [their] number one targets” to address (S1‐MP03). Parents agreed an integrative and collaborative approach with a support network of doctors and schools “would be ideal” (S2‐P04).

Another medical professional felt schools also had a responsibility in communicating medical and behavioural concerns to parents. It was felt teachers, once provided with knowledge, would be “be a bit more aware [to] spot the signs and inform those parents that might not be aware” (S1‐MP01). Parents agreed and thought that GPs should also be involved:

“If the doctors weren't supporting it and the schools and colleges weren't supporting it, you need everyone to work together and all know the actual situation and how it makes people feel.” (S2‐P11)

#### 3.2.4 Building awareness and “re‐educating” in schools

Participants felt that “if the awareness of chronic fatigue was a lot better, it would make it an easier experience [for the young person] to deal with in school” (S1‐A03). Parents and medical professionals identified school as an appropriate place for knowledge building to occur: “I think, for me it's education, better understanding in the community, and that has to start in schools” (S2‐P16).

Suggestions included using PHSE (Personal Health Social and Economic) lessons for educating pupils about ME/CFS, or an external speaker from the ME/CFS service to educate staff and students alike. Knowledge building was suggested to not only improve general awareness, but also specifically for teachers flagging concerns to healthcare services, and to understand their needs to provide appropriate support:

“*I think, really good communication with school and explaining that this is a chronic condition…Um and that we don't know when they're gonna get better.”* (S2‐MP14)

There were also discussions from parents and medical professionals about how best to go about “re‐educating schools” (S2‐P12) to include a person's “holistic needs not just academic” (S2‐P12), to “ensure the balance of life is correct for them and it's not just all around school” (S2‐MP14).

## 4 DISCUSSION

This incidental qualitative study highlights the importance of education and describes the realistic fears and hurdles for young people with ME/CFS remaining engaged in education. Families described concerns about the impact of missing school on their future and considered the role of school as important in recovery from ME/CFS. Some families discussed significant perceived barriers to accessing education in terms of a lack of practical and emotional support, which they felt negatively impacted adolescents' futures. Some participants, however, described good experiences with schools and felt supported to manage ME/CFS in the classroom. Adolescents valued feeling supported to manage their ME/CFS at school, or accessing education from home; their individual needs being recognised; and feeling confident about returning to school. Those who described good support from their school highlighted practical strategies implemented in the classroom, and it was thought that improved communication between education and healthcare would be useful.

A strength of this research is that combining two separate studies provides rich, multi‐perspective data (from adolescents, their parents/families, and a variety of medical professionals), and a larger sample size. Additionally, adolescents attended different schools in the region. Further, whilst neither study set out to explore experiences of schools, many families talked about how their interactions with schools and education were a defining feature of their ME/CFS journey. Participants from different demographics (ethnicity, age) and at different stages of recovery described similar experiences, illustrating the perceived issues with accessing education are unlikely limited to specific populations of adolescents with ME/CFS, rather, are ubiquitous.

The major limitation of this study was that neither study interviewed teachers so we cannot provide their perspective on supporting a pupil with ME/CFS. Furthermore, neither study specifically asked about helpful or unhelpful supportive school strategies, as ‘school’ was not a specific question in either study's topic‐guide. Therefore, we have limited data to offer practical strategic solutions and we cannot be certain that data saturation was achieved. Future research could use mixed methods to further explore the impact of different teaching strategies on the health of children and young people with ME/CFS. Additionally study 1 has a small sample size (due to the low numbers of ethnic minority children seen in the specialist ME/CFS service), and participants from both studies were recruited for specific populations or illness characteristics from one regional ME/CFS specialist service in England. Therefore, views may not be representative of adolescents with ME/CFS in other local authorities or schools.

Our findings, although from a small, regional sample, are consistent with other studies seeking feedback from young people on the importance of school. Educational engagement has been identified as crucial for ongoing well‐being and assisting positive outcomes in chronic illness (Hopkins et al., 2014; Lum et al., 2019) and adolescents with ME/CFS have described school at the centre of their recovery (Parslow et al., 2015). Similarly to other research, we found that some families have difficulties with schools recognising a ME/CFS diagnosis and implementing appropriate strategies for its management (Parslow et al., 2015; Tillett et al., 2000). Adolescents reported difficulties and disappointment when support or appropriate adaptations to meet their needs were not in place for them to remain in school, or engaged in educational activities from home. It is known that maintaining contact with school is important in ME/CFS (Nijhof et al., 2012; Rowe, 2019). Adolescents' main reported anxieties were around losing important social connections and not feeling able to fulfil their academic potential. This is corroborated with quantitative findings that young people with ME/CFS have reduced academic performance compared to healthy peers (Knight et al., 2018) and nearly 70% of young people with ME/CFS feel their illness significantly affects their career plans (Sankey et al., 2006).

Although a limited number of families in our study felt supported by school, the useful strategies they described focussed on allowing adolescents to remain engaged in their education at whatever level possible. They spoke about: rest opportunities; quiet ‘nurture’ rooms; a slower pace in lessons; and part‐time or reduced timetables. Suggestions for improved access to educational materials when adolescents are physically unable to attend school included: private tutors teaching outside of normal hours; remote access of lessons; or accessing recordings of lessons. Advances in technology, and lessons learnt from remote education in the COVID pandemic (Pokhrel & Chhetri, 2021), could be harnessed for teaching those with chronic illnesses such as through the use of ‘hybrid virtual classrooms’ (Klunder et al., 2022). Employing these strategies to maintain connections with school and friends is important because anxieties about returning to school can persist even after being recovered from ME/CFS (Parslow et al., 2015, 2017; Sankey et al., 2006). Participants' views in our study favouring a slow, graded return to school after a prolonged period away, corroborates with Sankey et al. (2006)'s research suggesting that a managed, graded return to school could overcome anxieties.

A prevalent frustration described by families was when they perceived that schools had misconceptions around ME/CFS, and lacked understanding of the illness and the needs of adolescents with ME/CFS. Similar to reports in other studies (Rowe, 2020), medical documentation sometimes resolved this and led to appropriately tailored support, but for many in our study, problems persisted even when medical evidence was provided. The families in our study who felt most supported by their school, felt strongly that a key reason for this was that their child's invidiual teachers not only had a good understanding of ME/CFS, but also had previous experience with ME/CFS either personally or from previous students. Families felt if there was more awareness of ME/CFS amongst staff and pupils, the experience of adolescents with ME/CFS would be improved. Suggestions to achieve improved awareness included using PHSE (Personal Health Social and Economic) lessons for educating pupils about ME/CFS, or an external speaker from the specialist ME/CFS service to educate staff and students alike.

Therefore, whilst it was recognised that school was an appropriate setting for improving awareness of ME/CFS, there likely needs to be additional considerations for how teachers can be supported and provided with the tools and knowledge (rather than just awareness) to meet the needs of pupils with ME/CFS. Whilst this study did not explore teachers' perspectives, we know from research in UK primary schools that teachers want more resources to increase their understanding of the condition and its management in the classroom (Brigden et al., 2021). Internet resources (www.wellatschool.org/chronic‐fatigue‐syndrome) (Well at School et al., 2020) exist to support awareness building about ME/CFS in schools, but as this study did not include teachers' views, an evaluation of the implementation of these resources or how to better support teachers' needs is unknown.

A further frustration described by families was that schools appeared to be bound by attendance targets and pressures from ‘higher ups’ in local authorities. This suggests that knowledge building in schools about the illness is clearly necessary, but not sufficient to meet the needs of both teachers and students, as teachers may have accountability requirements to educational authorities. Participants in our study recognised attendance pressures on schools and a need for involving local authorities to be involved in a multi‐way communication model of integrated care and felt better communication was required between healthcare, education and local authority bodies. A model of integrated care between schools, local authorities, and healthcare (ME/CFS services and GPs) has been shown to help young people with other chronic health conditions, including diabetes, cystic fibrosis and cancer, to function and remain socially engaged (St. Leger, 2014). Close liaison between health and education services can also aid in maintaining school attendance and reducing long‐term morbidity (Sankey et al., 2006). It should also be considered how ME/CFS services can provide educational outreach to schools and support teachers to help students. Participants in our study suggested a nominated individual should be involved from each body. Drawing on findings from other research, this could be a school nurse (Friedman et al., 2018; Tollit et al., 2018), a visiting teacher across multiple schools (Rowe & Fitzgerald, 1999) a named school doctor (Tillett et al., 2000), or someone in each health district with a special interest in ME/CFS (Sankey et al., 2006).

There is likely a role for medical professionals to advocate for adolescents with ME/CFS and their teachers at the local authority level to reduce the impost on classroom teachers and hence support students as well as providing information to schools in such a way as to make flexibility in requirements and reduced attendance acceptable. Future research should aim to capture the views of teachers to identify how ME/CFS specialist services can assist with implementing evidence‐based ME/CFS management and supportive strategies in the classroom, taking into account the complexities and demands of teachers roles and their accountability requirements to educational authorities.

## 5 CONCLUSION

This multi‐perspective paper provides rich data about adolescents' experiences with schools in the context of paediatric ME/CFS. Our findings highlight the importance of education, the barriers and anxieties that adolescents with ME/CFS face, as well as the complexities and demands on teachers and local authorities in meeting educational needs in chronic illness. We have elicited some positive strategies that adolescents described as helpful, and offer what families perceive is needed to improve care. These postitive strategies could be implemented alongside knowledge building initiatives, and an integrated model of communication between healthcare and education. Future research should focus on capturing the views of teachers and educational authorities to develop and evaluate these potential suggestions in consultation with healthcare professionals.

## AUTHOR CONTRIBUTIONS

CL (PhD, MA, BSc) and EC (MBChB, PhD) conceptualised and designed this paper, CL and PC (MBBS, BSc) conducted data collection and analysis for the two independent studies that this paper combines. All authors ‐ PC, CL, RP (PhD), JS (PhD), AL (PhD), JL (MBBS, MSc, BSc) and EC ‐ contributed to data analysis and interpretation included in this paper. PC and CL wrote the paper. All authors contributed to revisions and agreed the final draft.

## FUNDING INFORMATION

At the time of writing, PC was employed on the NIHR Academic Foundation Programme, and supported by the Elizabeth Blackwell Institute for Health Research, University of Bristol and the Wellcome Trust Institutional Strategic Support Fund (Grant code: 204813/Z/16/Z). CL received funding from a University of Bristol PhD studentship.

## CONFLICT OF INTEREST

Esther Crawley works as a non‐paid medical advisor for the Sussex and Kent ME society.

## Supporting information


Appendix S1

## Data Availability

The data that support the findings of this study are available from the corresponding author upon reasonable request.

## References

[hsc13942-bib-0001] Braun, V. , & Clarke, V. (2006). Using thematic analysis in psychology. Qualitative Research in Psychology, 3(2), 77–101. 10.1191/1478088706qp063oa

[hsc13942-bib-0002] Brigden, A. , Shaw, A. , Barnes, R. , Anderson, E. , & Crawley, E. (2020). “The child's got a complete circle around him”. The care of younger children (5–11 years) with ME/CFS. A qualitative study comparing families', teachers' and clinicians' perspectives. Health & Social Care in the Community, 28, 2179–2189. 10.1111/hsc.13029 32519359

[hsc13942-bib-0003] Brigden, A. , Shaw, A. , & Crawley, E. (2021). “It's a medical condition… you need to support as much as possible”: A qualitative analysis of teachers' experiences of chronic fatigue syndrome/myalgic encephalomyelitis (ME/CFS). BMC Pediatrics, 21(1), 1–9. 10.1186/s12887-020-02461-7 33397331 PMC7780629

[hsc13942-bib-0004] Crawley, E. , & Sterne, J. A. C. (2009). Association between school absence and physical function in paediatric chronic fatigue syndrome/myalgic encephalopathy. Archives of Disease in Childhood, 94(10), 752–756. 10.1136/adc.2008.143537 19001477

[hsc13942-bib-0005] Davies, S. , & Crawley, E. (2008). Chronic fatigue syndrome in children aged 11 years old and younger. Archives of Disease in Childhood, 93(5), 419–421. 10.1136/adc.2007.126649 18192312

[hsc13942-bib-0006] Department for Education . (2013). Ensuring a good education for children who cannot attend school because of health needs: Statutory guidance for local authorities. Retrieved from https://assets.publishing.service.gov.uk/government/uploads/system/uploads/attachment_data/file/269469/health_needs_guidance__‐_revised_may_2013_final.pdf

[hsc13942-bib-0007] Department for Education . (2015). Supporting pupils at school with medical conditions: Statutory guidance for governing bodies of maintained schools and proprietors of academies in England. Retrieved from https://assets.publishing.service.gov.uk/government/uploads/system/uploads/attachment_data/file/803956/supporting‐pupils‐at‐school‐with‐medical‐conditions.pdf

[hsc13942-bib-0008] Eccles, J. S. (1999). The development of children ages 6 to 14. The Future of Children, 9(2), 30–44. 10.2307/1602703 10646256

[hsc13942-bib-0009] Friedman, K. J. , Mattey, B. , & Newton, F. (2018). School nurses can improve the lives of students with Myalgic encephalomyelitis/chronic fatigue syndrome. NASN School Nurse, 33(6), 372–379. 10.1177/1942602X18795299 30222036

[hsc13942-bib-0010] Harland, M. R. , Parslow, R. , Anderson, N. , Byrne, D. , & Crawley, E. (2019). Paediatric chronic fatigue syndrome patients' and parents' perceptions of recovery. BMJ Paediatrics Open, 3(1), e000525. 10.1136/bmjpo-2019-000525 32500105 PMC7245384

[hsc13942-bib-0011] Hopkins, L. , Green, J. , Henry, J. , Edwards, B. , & Wong, S. (2014). Staying engaged: The role of teachers and schools in keeping young people with health conditions engaged in education. The Australian Educational Researcher, 41, 25–41.

[hsc13942-bib-0012] Jelbert, R. , Stedmon, J. , & Stephens, A. (2010). A qualitative exploration of adolescents' experiences of chronic fatigue syndrome. Clinical Child Psychology and Psychiatry, 15(2), 267–283. 10.1177/1359104509340940 20179018

[hsc13942-bib-0013] Klunder, A. , Saab, N. , & Admiraal, W. (2022). A teacher perspective on using a hybrid virtual classroom for students with a chronic illness in mainstream primary and secondary schools. Technology, Pedagogy and Education, 1–16. 10.1080/1475939X.2022.2033824

[hsc13942-bib-0014] Knight, S. J. , Politis, J. , Garnham, C. , Scheinberg, A. , & Tollit, M. (2018). School functioning in adolescents with chronic fatigue syndrome. Frontiers in Pediatrics, 6, 302–310. 10.3389/fped.2018.00302 30460211 PMC6232780

[hsc13942-bib-0015] Lim, E. J. , Ahn, Y. C. , Jang, E. S. , Lee, S. W. , Lee, S. H. , & Son, C. G. (2020). Systematic review and meta‐analysis of the prevalence of chronic fatigue syndrome/myalgic encephalomyelitis (ME/CFS). Journal of Translational Medicine, 18(1), 1–15. 10.1186/s12967-020-02269-0 32093722 PMC7038594

[hsc13942-bib-0016] Lum, A. , Wakefield, C. E. , Donnan, B. , Burns, M. A. , Fardell, J. E. , Jaffe, A. , Kasparian, N. A. , Kennedy, S. E. , Leach, S. T. , Lemberg, D. A. , & Marshall, G. M. (2019). School students with chronic illness have unmet academic, social, and emotional school needs. School Psychology, 34(6), 627–636. 31697148 10.1037/spq0000311

[hsc13942-bib-0017] Maslow, G. R. , Haydon, A. , McRee, A. L. , Ford, C. A. , & Halpern, C. T. (2011). Growing up with a chronic illness: Social success, educational/vocational distress. Journal of Adolescent Health, 49(2), 206–212. 10.1016/j.jadohealth.2010.12.001 PMC3414253 21783055

[hsc13942-bib-0018] NICE . (2007). Chronic fatigue syndrome/myalgic encephalomyelitis (or encephalopathy): Diagnosis and management of ME/CFS in adults and children (NICE guidelines CG53). (Vol. CG53).

[hsc13942-bib-0019] Nijhof, S. L. , Bleijenberg, G. , Uiterwaal, C. S. , Kimpen, J. L. , & van de Putte, E. M. (2012). Effectiveness of internet‐based cognitive behavioural treatment for adolescents with chronic fatigue syndrome (FITNET): A randomised controlled trial. The Lancet, 379(9824), 1412–1418. 10.1016/S0140-6736(12)60025-7 22385683

[hsc13942-bib-0020] Parslow, R. , Harris, S. , Broughton, J. , Alattas, A. , & Crawley, E. (2017). Children's experiences of chronic fatigue syndrome/myalgic encephalomyelitis (ME/CFS): A systematic review and meta‐ethnography of qualitative studies. BMJ Open, 7(1), e012633. 10.1136/bmjopen-2016-012633 PMC5253584 28087544

[hsc13942-bib-0021] Parslow, R. , Patel, A. , Beasant, L. , Haywood, K. , Johnson, D. , & Crawley, E. (2015). What matters to children with ME/CFS? A conceptual model as the first stage in developing a PROM. Archives of Disease in Childhood, 100(12), 1141–1147. 10.1136/archdischild-2015-308831 26453575 PMC4680202

[hsc13942-bib-0022] Pokhrel, S. , & Chhetri, R. (2021). A literature review on impact of COVID‐19 pandemic on teaching and learning. Higher Education for the Future, 8(1), 133–141.

[hsc13942-bib-0023] QSR International Pty Ltd . (2015). NVivo (Version 11). Retrieved from https://www.qsrinternational.com/nvivo‐qualitative‐data‐analysis‐software/home

[hsc13942-bib-0024] QSR International Pty Ltd . (2018). NVivo (Version 12). Retrieved from https://www.qsrinternational.com/nvivo‐qualitative‐data‐analysis‐software/home

[hsc13942-bib-0025] Rangel, L. , Garralda, M. , Leven, M. , & Roberts, H. (2000). The course of severe chronic fatigue syndrome in childhood. Journal of the Royal Society of Medicine, 93(3), 129–134. 10.1177/014107680009300306 10741312 PMC1297949

[hsc13942-bib-0026] Rowe, K. (2019). Long term follow up of young people with chronic fatigue syndrome attending a pediatric outpatient service. Frontiers in Pediatrics, 7, 21. 10.3389/fped.2019.00021 30847333 PMC6393360

[hsc13942-bib-0027] Rowe, K. (2020). Paediatric patients with myalgic encephalomyelitis/chronic fatigue syndrome value understanding and help to move on with their lives. Acta Paediatrica, 109(4), 790–800. 10.1111/apa.15054 31854020 PMC7154625

[hsc13942-bib-0028] Rowe, K. , & Fitzgerald, P. (1999). Educational strategies for chronically ill students: Chronic fatigue syndrome. The Australian Educational and Developmental Psychologist, 16(2), 5–21.

[hsc13942-bib-0029] Sankey, A. , Hill, C. M. , Brown, J. , Quinn, L. , & Fletcher, A. (2006). A follow‐up study of chronic fatigue syndrome in children and adolescents: Symptom persistence and school absenteeism. Clinical Child Psychology and Psychiatry, 11(1), 126–138. 10.1177/1359104506059133 17087490

[hsc13942-bib-0030] St Leger, P. (2014). Practice of supporting young people with chronic health conditions in hospital and schools. International Journal of Inclusive Education, 18(3), 253–269. 10.1080/13603116.2012.679320

[hsc13942-bib-0031] Tillett, A. , Glass, S. , Reeve, A. , & Burt, A. (2000). Provision of health and education services in school children with chronic fatigue syndrome. Ambulatory Child Health, 6, 83–89. 10.1046/j.1467-0658.2000.00065.x

[hsc13942-bib-0032] Tollit, M. , Politis, J. , & Knight, S. (2018). Measuring school functioning in students with chronic fatigue syndrome: A systematic review. Journal of School Health, 88(1), 74–89. 10.1111/josh.12580 29224219

[hsc13942-bib-0033] Well at School , Crawley, E. , Parslow, R. , Loades, M. , & Brigden, A. (2020). *Helping Children With Medical And Mental Health Conditions Get The Best Out Of School: CHRONIC FATIGUE SYNDROME/ME* (Well at School). Retrieved from www.wellatschool.org/chronic‐fatigue‐syndrome

[hsc13942-bib-0034] World Health Organisation . (2016). The global strategy for women's, children's and adolescents' health (2016‐2030). *Every Women Every Child*. Retrieved from https://www.everywomaneverychild.org/global‐strategy/#sect2

