# Supplementary material for: The importance of school in the management of Myalgic Encephalomyelitis/Chronic Fatigue Syndrome (ME/CFS): issues identified by adolescents and their families
Source: Health Soc Care Community. 2022 Aug 22;30(6):e5234–44. doi: 10.1111/hsc.13942 (PMC10087786; doi:10.1111/hsc.13942)
Supplement: Supplementary file 1 — Appendix S1 [file HSC-30-e5234-s001.docx]

1. Appendix: Topic Guides

**Study 1’s Example Topic Guide – Family Interview.**

This topic guide details interview topics to be covered. The interviewer might also ask additional questions to clarify information.

**Prior to the start of the interview:**

The aim of this study is to understand children's experiences of disabling fatigue and access to medical services. This interview will be recorded, during transcription any details will be made confidential and informed consent will be carried out.

**Part A: Information about illness**

- What are your family member's main symptoms?
  - Do they have any other symptoms?
- How long have they experienced disabling fatigue?
- How many days a week are they able to attend school/college?
- How has the family changed since the child/adolescent has been unwell?

**Part B: Information about medical care**

- Do you (and your family) have a good relationship with a GP?
  - What are your experiences accessing medical care for yourself and your family members?
- Has your family member visited a GP about your symptoms / how many times have they visited a GP about the symptoms?
- Have they received a diagnosis?
- What was their experience like visiting a GP?
- Have you consulted anyone else about their symptoms, e.g. a herbalist, spiritual healer?

**Part C: Information about community**

- Do you know anyone else in your community who experiences similar symptoms?
- How is fatigue viewed in your community?

**Part D: Close**

- Are there any issues you would like to raise that we have not talked about?
- Thank you for your time and contribution to this study.

**Prompts**: The following prompts may be used by the interviewer to explore certain answers in more detail:

- Can you describe that?
- Could you give me an example?
- Why did you do that?
- Could you tell me more about that?

**Study 2’s Example Topic Guide: Adolescent Interview.**

This topic guide details the areas that will be covered in the qualitative interviews. The interviewer may ask additional questions during the interview to clarify information. The questions may be minimally adapted throughout the process of interviewing as observations or alternate questions arise.

1. **Introductions**

Explain we are talking to children and young people with CFS/ME who have not yet fully recovered after their initial treatment to help find alternative treatments.

1. **Interview topics**
   1. **Treatment to date**

**Can you tell me about the treatment you have had for your CFS so far?**

Prompts: Who have you seen - a physio? psychologist?

Note: Be mindful that patients might not be able to name the treatment but will be able to say who they've seen (psych, physio etc.) and will be able to comment on some content (e.g. activity diaries etc.). They may also tell you about things they did before the Bath service so may need to specify*.*

What did you think of it?

What did you like? Why did you like that?

What did you not like? Why did you not like that?

What was helpful? Why?

Place (in person/remote/need for travel)?

Time of day?

Structured (not enough/hands-off or too much/hands-on)?

**What are the things that have helped you feel better?**

Prompts: Good/favourite?

Bad/never want to do again?

- 1. **Needs from future treatments**

**How do you feel about your recovery?**

Prompt: What kind of feelings come up when we talk about recovery?

**How can we make your treatment better?**

Prompts: What's missing from current treatment?

What needs to change?

How can we help you move closer to recovery?

How can your therapist best support you to make those changes?

Going forward, what do you think would help?

What do you need to work on next?

What's stopping you/getting in the way/holding you back from getting better quicker?

**What sort of treatment would work best for you?**

Prompts: Heard or read about things you want to try?

What is main thing you need help with to get better?

What do you think will help you get better from fatigue?

- 1. **Perceptions of ACT**

**Explain about ACT** **+** **vignette.**

ACT stands for Acceptance and Commitment Therapy. It is a type of psychological treatment which is helpful for lots of people. ACT looks at the way you feel, how you are thinking, and how this affects what you do. It's a bit like CBT. But a big part of ACT is that it recognises it is normal to have tricky thoughts and feelings sometimes, and that you do not have to get rid of them. ACT suggests that trying to get rid of them can cause more problems. Instead, ACT helps you learn to still do the things you want, even when it's hard. In ACT you also think about your values; the things in life that are really important to you.

**What do you think about the ACT treatment James is having?**

Prompt: Have you heard of it before?

What are your first thoughts about it? Good? Bad?

Would you want to try ACT? Which parts would you want to try?

The way ACT focuses on your values rather than challenging thoughts?

*Note: May need to explain VALUES. Values are things that are important to you in life. The bigger reason why you do the things you do. Example: a goal is going to school but a value is why you are going to school. For your education or because you want to learn things, want to go to university or want a job.*

The idea of stepping away from thoughts (cognitive diffusion)?

The idea of seeing things can be bad but still trying to achieve your goals?

Do you think it would be helpful for you to think about these things?

Do you think it would help other people with fatigue?

What about it do you think would be helpful?

**Explain Trial.**

We're thinking about delivering ACT as part of a trial. A trial is a type of research study where we want to test 2 treatments. In this case, we would be trialling ACT against the normal supportive pathway that is currently available for children who have not fully recovered after 1 year. We would assign each child to either ACT or the supportive pathway. This would be random and you would not be able to choose which pathway you receive.

As researchers and doctors we know a lot about how to do trials in an ideal world, however, we need to make sure it suits patients and their parents because we do not know how it feels to live with CFS/ME on a daily basis and what your needs and wants are. We need to make sure it's a realistic and feasible trial. That's why we ask your honest opinions on things like this.

**What type of things do we as researchers and doctors need to think about when using ACT as a treatment for you?**

Prompt: What would you like to know more about before having ACT?

Would you want to try it after/before other treatment?

Anything you do not like the sound of?

**If we do this trial, how do you feel about taking part?**

Prompt: How should we ask children like you to take part in ACT?

What would you think about when deciding whether or not to take part?

Barriers to participating?

Who/what would influence you? Therapist? Family? GP? Others?

Would you like face-to-face or skype sessions?

How many sessions would you like?

What would you expect from the trial?

Is there anything we have not thought of that you would want us to know about trialling this treatment?

Any recommendations for how to do trial?

**If we do this trial, how do you feel about being randomised to ACT or supportive path?**

Prompt: What makes you say that answer?

1. **Close**

**Is there anything else you would like to tell me?**

Thank participant for taking part.
